# Characterization and Categorization of Various Human Lower Limb Movements Based on Kinematic Synergies

**DOI:** 10.3389/fbioe.2021.793746

**Published:** 2022-01-20

**Authors:** Bo Huang, Wenbin Chen, Jiejunyi Liang, Longfei Cheng, Caihua Xiong

**Affiliations:** State Key Laboratory of Digital Manufacturing Equipment and Technology, Institute of Rehabilitation and Medical Robotics, Huazhong University of Science and Technology, Wuhan, China

**Keywords:** categorization, lower limb, locomotion, kinematic coordination, principal component analysis, cluster analysis, rehabilitation, robotics

## Abstract

A proper movement categorization reduces the complexity of understanding or reproducing human movements in fields such as physiology, rehabilitation, and robotics, through partitioning a wide variety of human movements into representative sub-motion groups. However, how to establish a categorization (especially a quantitative categorization) for various human lower limb movements is rarely investigated in literature and remains challenging due to the diversity and complexity of the lower limb movements (diverse gait modes and interaction styles with the environment). Here we present a quantitative categorization for the various lower limb movements. To this end, a similarity measure between movements was first built based on limb kinematic synergies that provide a unified and physiologically meaningful framework for evaluating the similarities among different types of movements. Then, a categorization was established *via* hierarchical cluster analysis for thirty-four lower limb movements, including walking, running, hopping, sitting-down-standing-up, and turning in different environmental conditions. According to the movement similarities, the various movements could be divided into three distinct clusters (cluster 1: walking, running, and sitting-down-standing-up; cluster 2: hopping; cluster 3: turning). In each cluster, cluster-specific movement synergies were required. Besides the uniqueness of each cluster, similarities were also found among part of the synergies employed by these different clusters, perhaps related to common behavioral goals in these clusters. The mix of synergies shared across the clusters and synergies for specific clusters thus suggests the coexistence of the conservation and augmentation of the kinematic synergies underlying the construction of the diverse and complex motor behaviors. Overall, the categorization presented here yields a quantitative and hierarchical representation of the various lower limb movements, which can serve as a basis for the understanding of the formation mechanisms of human locomotion and motor function assessment and reproduction in related fields.

## Introduction

The human lower limb shows extraordinary motor ability in daily living, which is indispensable for humans in independent living. Through flexible use of the lower limb, humans can move in various gait styles and interact with diverse environmental conditions to cope with different requirements of activities of daily living. In the past, to understand, imitate, repair, or enhance the motor ability of the human lower limb, a lot of research has been done (Tucker et al., 2015; Young and Ferris, 2017; Koyama and Yamauchi, 2018; Price et al., 2019; Yao et al., 2019; Sun et al., 2020; Rodriguez-Fernandez et al., 2021). However, the enough motor dexterity of the human lower limb, as indicated by the diversity of their movement styles, brings challenges to the study of the lower limb movements. In this context, it can be predicted that the complexity of this challenging problem can be reduced by building a categorization for the wide variety of lower limb movements, which can partition the many lower limb movements into a series of small, representative, and homogenous sub-motion categories based on their similarities or differences.

Partitioning or categorization of the lower limb movements is useful in several fields (Papageorgiou et al., 2019; Schambra et al., 2019; Stival et al., 2019). In physiology, it can provide new insights into understanding the formation mechanisms of the lower limb movements. The formation of a category consisting of many different movements can uncover several similar control strategies employed by these movements in the category, which can help clarify whether there exist conserved control strategies across the various movements. In contrast, some potential mechanisms underlying the flexibility and plasticity of the human motor system can also be uncovered by the finding and comparison of different categories. In rehabilitation, a categorization can facilitate the assessment of motor function by subdividing the many movements into a few meaningful and manageable sub-motion categories and can avoid the risk of neglecting some important categories that have an impact on the assessment and subsequent rehabilitation treatments. Meanwhile, such categorization can also allow us to customize a standardized rehabilitation program for each category in order to achieve better treatment outcomes. In robotics, a categorization can promote the development of artificial limbs imitating or enhancing human motor ability (e.g., prostheses and exoskeletons). The categories and their movement characteristics can provide references for comparing the performance of the artificial limbs with the human limb, thereby encouraging the development of better mechanical or control systems. In practice, motivated by the advantages of the categorization, researchers have built different taxonomies or classification systems for different types of human movements, such as the taxonomies of hand grasps (Feix et al., 2016; Stival et al., 2019), whole-body support poses (Borras et al., 2017), and activities of daily living of upper limb (Gloumakov et al., 2020a; Gloumakov et al., 2020b), and the classification systems of normal walking (Vardaxis et al., 1998; Simonsen and Alkjaer, 2012), normal running (Liebl et al., 2014; Phinyomark et al., 2015), and pathological walking or running (Kuntze et al., 2018; Jauhiainen et al., 2020). However, to our knowledge, a quantitative categorization for the various lower limb movements has not been established to date. Given the diversity of human gait modes and interaction styles with the external environment, there are several questions that remain to be resolved for establishing the categorization: which movements are similar to each other and how to quantify the similarities among different types of lower limb movements.

To measure the similarity between movements, a key question is to select unified, informative, and quantitative movement descriptors which can characterize the various lower limb movements. In previous studies, to identify the subsets or categories underlying human gaits, the nature of gaits is usually described by some discrete gait parameters (Vardaxis et al., 1998; Mulroy et al., 2003; Simonsen and Alkjaer, 2012; Jauhiainen et al., 2020), such as phasic, spatiotemporal gait parameters (e.g., speed, stride length, cadence, and duty factor), or some critical kinematic and kinetic parameters (e.g., peak joint angles, moments, and powers, or joint angles, moments, and powers at specific events or phases in walking or running gaits). For example, walking and running, the terminologies describing the two most common lower limb movement modes, are usually differentiated by the duty factor (the fraction of the stride duration when each foot is on the ground) (Kram et al., 1997; Segers et al., 2006; Fihl and Moeslund, 2007). Likewise, through examining the similarity of kinematic and kinetic parameters, more than one category requiring different movement strategies has also been identified in normal walking, rather than only a single normative template of walking pattern as is often assumed (Vardaxis et al., 1998; Simonsen and Alkjaer, 2012). Without a doubt, these gait descriptors have provided a good basis for distinguishing walking or/and running gaits. However, only partial information is provided by these gait descriptors, which may obscure many other subtle features underlying the human lower limb movements (Kuntze et al., 2018; Sawacha et al., 2020). Moreover, part of the movement descriptors are suitable for characterizing walking and running but not for some other lower limb movements achieved by humans (e.g., sit-to-stand and turning in place) (Vardaxis et al., 1998; Etnyre and Thomas, 2007; Prakash et al., 2018).

In particular, studies in the field of motor control show that the many joint motions in the process of the limb movements are not independent of each other but constrained by the nervous system (Borghese et al., 1996; St-Onge and Feldman, 2003; Grillner and El Manira, 2020), which cannot be uncovered by the discrete and independent gait parameters mentioned above. Specifically, to generate a complex behavior, the joint motions are coordinated by the nervous system to bend or stretch together as several basic units or synergies. Then, the complex behavior can be constructed rapidly and efficiently through the combination of a small number of synergies. Inspired by the existence of the synergies, it can be argued that it is necessary to consider the coordination among joints when characterizing the lower limb movements, rather than only considering the characteristics of individual joints separately. More importantly, the joint synergies have been found in different lower limb movements, including various cyclical and non-cyclical movements (e.g., squats, walking, going up or down a step, and running) (St-Onge and Feldman, 2003; Hicheur et al., 2006; Moro et al., 2012). Similarly, a planar covariation law of intersegmental coordination is also found in human locomotion (Borghese et al., 1996; Ivanenko et al., 2007), where the temporal changes in the elevation angles of lower limb segments (thigh, shank, and foot) are found to be covariant along an attractor planar. This planar covariation law has been observed in human running (Hicheur et al., 2006), hopping (Ivanenko et al., 2007), crawling (MacLellan et al., 2017), and various walking tasks, such as level walking (Borghese et al., 1996; Bianchi et al., 1998; Lacquaniti et al., 1999; Dominici et al., 2011; Catavitello et al., 2018; Gueugnon et al., 2019), walking on slopes (Noble and Prentice, 2008; Dewolf et al., 2018), and backward walking (Grasso et al., 1998). Therefore, the synergies can be expected to provide a new, unified, and biologically meaningful framework for describing different types of lower limb movements and examining their similarities. In addition, many studies suggest that the joint synergies play a role in the motor function assessment (e.g., abnormal joint coordination) and treatment planning in rehabilitation (Jarrasse et al., 2014; Ting et al., 2015). Likewise, our previous work finds that the prostheses and exoskeleton developed based on the joint synergies are able to reproduce human-like motor ability (i.e., the reproduction of human-like joint angle trajectories) (Chen et al., 2015; Xiong et al., 2016; Liu et al., 2018). Taken together, it can also be expected that the movement representation based on the synergies will provide new and complementary insights into understanding, classification, and reproduction of the lower limb movements in related fields, compared with traditional gait descriptors.

This paper proposes a measure index of movement similarity through synergy-based movement representation and presents a quantitative categorization for a variety of human lower limb movements. Taking into account the diversity of the human lower limb movements, we collected motion data from the motor tasks which to some extent represent the versatile motor ability of the lower limb in daily living (Kuehne et al., 2011; Mandery et al., 2016). Then, we applied cluster analysis to identify the primary and representative categories underlying the various lower limb movements according to synergy-based movement similarities. Finally, we analyzed the coordination features of the movement categories in the categorization, in order to uncover category-specific control strategies in each category and the set of available and typical synergies used by humans.

## Materials and Methods

### Participants

The human lower limb can achieve diverse movements in daily living. To build a comprehensive and representative categorization for the lower limb movements and explore their similarities and differences, a motion dataset from previous work of the authors (Huang et al., 2021a; Huang et al., 2021b) was analyzed in this study. Nine healthy male subjects (age: 23.0 ± 1.0 years; weight: 64.0 ± 6.1 kg; height: 173.1 ± 4.1 cm; mean ± s.d.) participated in the experiment [no differences in the kinematic coordination are found between men and women in the past (Hicheur et al., 2006; Chow and Stokic, 2015)]. The sample size was chosen based on previous studies (St-Onge and Feldman, 2003; Ivanenko et al., 2007; Funato et al., 2010; Catavitello et al., 2018; Dewolf et al., 2018). The experimental protocol was approved by the Chinese Ethics Committee of Registering Clinical Trails. All the subjects provided consent prior to participation.

### Experimental Procedure

**FIGURE 1:**
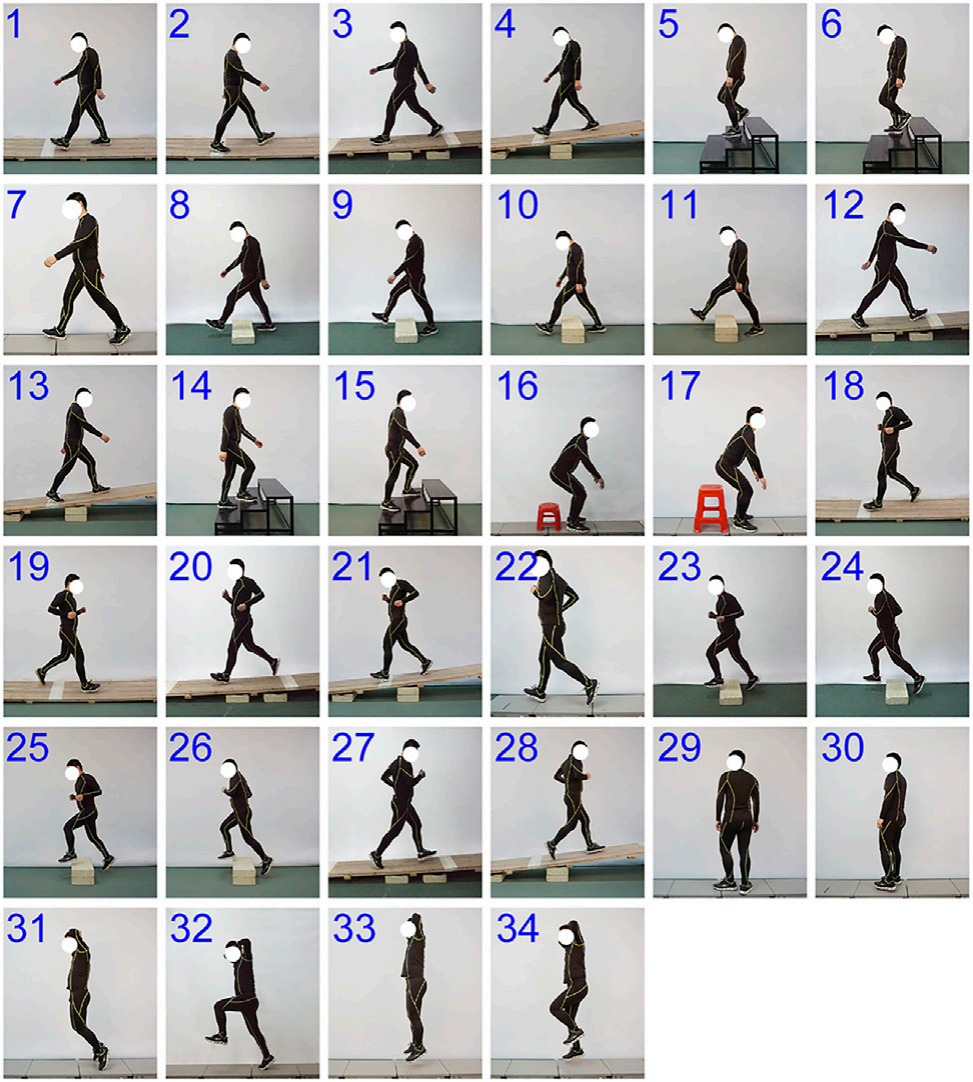
Motor tasks explored in this study. Thirty-four motor tasks are analyzed in this study (Nos. 1–15: walking tasks, Nos. 16 and 17: sitting-down-standing-up tasks, Nos. 18–28: running tasks, Nos. 29 and 30: turning in place tasks, and Nos. 31–34: hopping tasks). Adapted from Huang et al., 2021a.

In this experiment, five basic motor modes which can represent the versatile motor ability of the lower limb were included: walking (Nos. 1–15; Figure 1), sitting-down-standing-up (chair height: 30.2 or 42.7 cm; Nos. 16 and 17), running (Nos. 18–28), turning in place (Nos. 29 and 30), and hopping (hopping forward or in place on two legs or only the right leg; Nos. 31–34) (Kuehne et al., 2011; Mandery et al., 2016). Moreover, considering the effect of natural environment constraints on the limb movements, the subjects were asked to walk or run under five typical ground conditions: level ground (7 m walkway; Nos. 7 and 22), cross slopes (incline angle with respect to the level walkway: ± 14.5°; the “+” represented that the left side of the walkway was higher than the right side: Nos. 2 and 19, the “–” represented the opposite case: Nos. 1 and 18), longitudinal slopes (incline angle: ± 2.6° and ±6°; the “+” represented upslope: Nos. 12, 13, 27, and 28, the “−” represented downslope: Nos. 3, 4, 20, and 21), obstacles (width: 30 cm; height: 10 or 20 cm; Nos. 8–11 and Nos. 23–26), and stairs (riser: 15 cm; tread: 30 cm; Nos. 5, 6, 14, and 15). In total, motion data from thirty-four different motor tasks were used in this study. For all the motor tasks, the subjects were asked to choose their preferred speeds and cadences in order to perform these tasks in a natural way. Moreover, all the motor tasks were recorded three times.

The Vicon Motion Capture System (Oxford Metrics, United Kingdom) with 10 cameras was used to record human kinematic data at a sampling frequency of 100 Hz. 20 reflective markers (diameter: 14 mm) were attached to the body landmarks of the lower limbs according to the Plug-in Gait model provided by the Nexus software (Oxford Metrics, United Kingdom). Two additional calibration markers were attached to the left and right medial malleoli during a static trial, which had the subjects stand still. During hopping, the ground reaction forces were recorded by four AMTI force plates (60 cm × 40 cm; sampling frequency: 1,000 Hz; Advanced Mechanical Technology Inc., United States) placed in the middle of the walkway and along the motion direction.

### Data Pre-processing

Kinematic data (i.e., hip, knee, and ankle joint angles) were calculated by the Plug-in Gait model after the trajectories of markers were filtered by a Woltring filter with a mean-squared error of 20 mm^2^ (Woltring, 1986). The ground reaction forces were low-pass filtered with a fourth-order Butterworth filter (cutoff frequency: 25 Hz).

The foot contact event was determined by the timing when the speed of the heel marker was less than 0.4 m/s during walking and running (Noble and Prentice, 2008) or when the vertical component of the ground reaction forces was greater than 7% of the body weight during hopping (Ivanenko et al., 2007). For walking (except walking up and down stairs), running, and hopping, the motion data over a gait cycle (the time period between two successive foot contacts of the same foot) were retained for each trial. For walking or running over an obstacle, the motion data over a gait cycle that could cover the entire process of stepping over the obstacle were retained. In other words, the motion data between two successive left foot contacts were retained when stepping over the obstacle starting with the right leg, vice versa. For walking up and down stairs, sitting-down-standing-up, and turning in place, the data from the beginning time of the movement to the ending time were retained. After the motion data were selected, the joint angle sequences of each trial were resampled to 200 points using cubic spline interpolation.

Here the coordination patterns among six joint motions of the right lower limb were studied and used to describe the characteristics of the lower limb movements: hip flexion/extension (H f/e), hip adduction/abduction (H a/a), hip rotation (H rot), knee flexion/extension (K f/e), ankle plantarflexion/dorsiflexion (A p/d), and ankle rotation (A rot). Flexion, adduction, internal rotation, and dorsiflexion were defined as positive values in this study. The posture during the static trial (standing still) was set as initial posture so that the mean joint angles during the static trial were subtracted from the joint angle values during each dynamic trial. For each trial, the joint angles of the lower limb were presented as a data matrix $Q=\left[\begin{array}{c} q_{1}\cdots q_{i}\cdots q_{200} \end{array}\right]$ , $q_{i}\in {\mathbb{R}}^{6\times 1}$ represents the posture of the lower limb at the *i*th moment. To comprehensively characterize the limb movement patterns for a specific motor task, the data from all the three trials were pooled together as a data matrix $Q_{t}\in {\mathbb{R}}^{6\times 600}$ for each subject.

### Similarity Between Movements

As suggested by the studies in the kinematic synergies, limb joint motions in a motor task can be decomposed into a series of kinematic synergies and reconstructed by their linear combination. The kinematic synergies thus provided a framework for characterizing the various lower limb movements and were used to quantify the similarity between the movements in this study. Following this, we first extracted the kinematic synergies of each of the thirty-four tasks by using principal component analysis on the data matrix $Q_{t}$ consistent with previous studies (St-Onge and Feldman, 2003; Ivanenko et al., 2007). In this way, original joint motions could be represented as $q_{i}-{\bar{q}}_{i}=\overset{6}{\underset{j=1}{\sum }}c_{ji}s_{j}$ . ${\bar{q}}_{i}$ is the average of $q_{i}$ . $s_{j}$ is the *j*th synergy equal to the eigenvector of the covariance matrix of the joint motions with the *j*th largest eigenvalue, and the elements of $s_{j}$ (weightings) represent the contributions of the joint motions to the synergy [the absolute value of a weighting above 0.25 was defined as indicating significant contribution (Gracia-Ibanez et al., 2020)]. The synergies are ordered according to the variance explained by each synergy from largest to smallest. Thus, the proportion of the total variance explained by the *j*th synergy is $PVE_{j}=\lambda_{j}/\overset{6}{\underset{r=1}{\sum }}\lambda_{r}$ ( $\lambda_{j}$ is the variance explained by the *j*th synergy and equal to the *j*th largest eigenvalue of the covariance matrix of the joint motions). $\;c_{ji}$ (recruitment coefficient of the synergy) represents the contribution of the synergy to the original joint motion patterns at the *i*th moment.

Then, to measure the similarity between a pair of motor tasks, the similarity between two synergies was first quantified by the absolute value of their scalar product. Two synergies were considered significantly similar if their similarity >0.7 (Tresch et al., 1999; Torres-Oviedo and Ting, 2010). Then, based on the synergy similarities and taking into account the different contribution rates of the synergies, a similarity index (*SI*) between two motor tasks (e.g., the *m*th and *n*th tasks) was defined:

$$SI=\overset{6}{\underset{j=1}{\sum }}\left(\frac{1}{2}\left(PVE_{j}^{m}+PVE_{j}^{n}\right)\left|s_{j}^{m}\cdot s_{j}^{n}\right|\right)$$

Obviously, the similarity index ranges from 0 to 1, and a smaller value indicates a higher difference between two tasks.

### Identification of Movement Categories

Agglomerative hierarchical clustering method was used to identify the categories of the lower limb movements according to the movement similarities. Before the start of cluster analysis, the similarities were measured among all the tasks and averaged across the subjects. To form the clusters, the similarity between two new clusters at each combination stage was determined by the average linkage algorithm (Johnson and Wichern, 2007).

Hierarchical clustering method always results in a number of possible cluster solutions. In this context, Mojena stopping rule (lower-tail method) was applied to determine the number of clusters in the final solution (Mojena, 1977; Simonsen and Alkjaer, 2012). According to this stopping rule, the optimal cluster solution is the solution corresponding to the first cluster combination stage *i*, which satisfies the inequality $\alpha_{i+1}<\bar{\alpha }-ks_{\alpha }$ , where $\alpha_{i+1}$ is the fusion level in the stage *i+1* with *33−i* clusters (i.e., the similarity determined by the average linkage algorithm); $\bar{\alpha }$ and $s_{\alpha }$ are the mean and standard deviation of the *α* distribution, respectively; *k* is the standard deviate and is set to 1.25 according to the recommendation of a simulation study (Milligan and Cooper, 1985).

### Core Synergies of Each Category

After movement clusters were identified, the synergies for a cluster representing the overall coordination characteristics of the cluster (called core synergies of the cluster in the following sections) were extracted from the motion data of all the motor tasks within the cluster. The data from the motor tasks in the cluster were pooled together as an entire data matrix in each subject, and then the synergies for the cluster were extracted. In this stage, to further examine the main differences between clusters, we selected the minimum number of the primary synergies which could capture the main movement variation of the motor tasks in each cluster. To this end, two criterions were used (global and local criterions). First, the main synergies of a cluster could account for more than 90% of the overall movement variance of all the motor tasks in the cluster (Courtine and Schieppati, 2004). Second, the main synergies of a cluster could account for more than 90% of the movement variance of each motor task in the cluster. The stringent local criterion ensured that the characteristics of each task in a cluster could be well described. Following this, the differences between the clusters were examined based on the core synergies using the absolute value of the scalar product of the core synergies.

### Statistical Analysis

To examine the subtle differences between two core synergies, the difference in the weighting of a joint motion between two core synergies is further compared using a two-tailed paired *t*-test. Sample normality was verified using the Lilliefors test. The significance level was set at *α* = 0.05. Similarly, for a specific synergy, the difference in the weighting between two joint motions was also compared. All statistical analyses were performed using MATLAB R2017a (Mathworks, Natick, MA, United States).

## Results

In brief, the speeds of all walking tasks (except for walking upstairs and walking downstairs), all running tasks, and all forward hopping tasks ranged from 1.06 ± 0.13 m/s (No. 11) to 1.27 ± 0.13 m/s (No. 4; mean ± s.d. across all the trials performed by all the subjects), from 1.94 ± 0.31 m/s (No. 19) to 2.13 ± 0.25 m/s (No. 22), from 1.46 ± 0.32 m/s (No. 31) to 1.69 ± 0.30 m/s (hopping forward on the right leg; No. 32), respectively. The hopping frequency ranged from 1.40 ± 0.29 Hz (No. 31) to 1.96 ± 0.53 Hz (hoping in place on the right leg; No. 34). The duration of movement was 3.38 ± 0.44 s in walking downstairs, 3.66 ± 0.31 s in walking upstairs, 3.85 ± 0.92 s in sitting-down-standing-up, and 4.76 ± 0.59 s in turning.

### Similarities and Categories of the Lower Limb Movements

**FIGURE 2:**
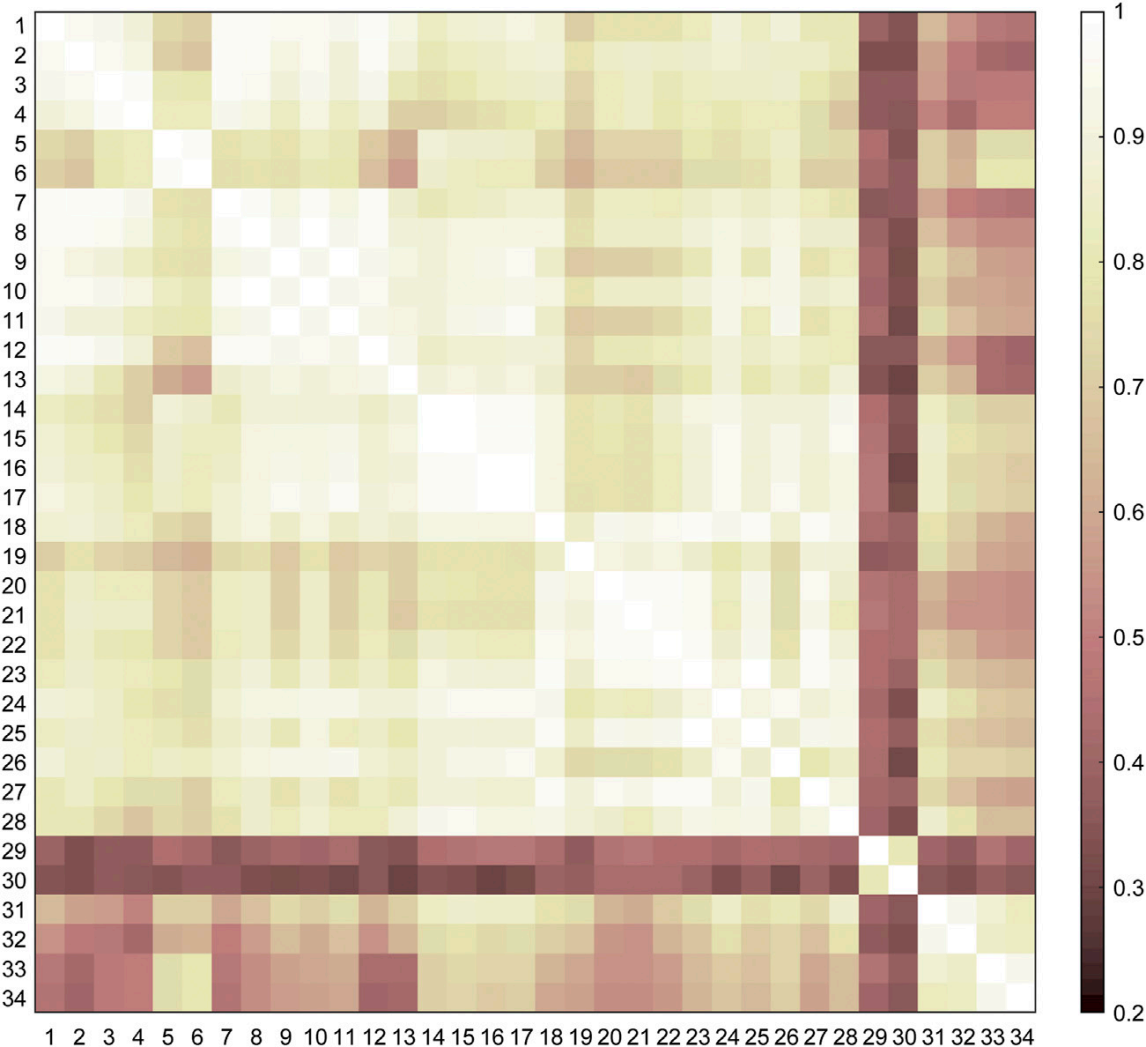
Similarities among the motor tasks. The similarities are the averages across the subjects (*n* = 9) and are used as the input of cluster analysis.

**FIGURE 3:**
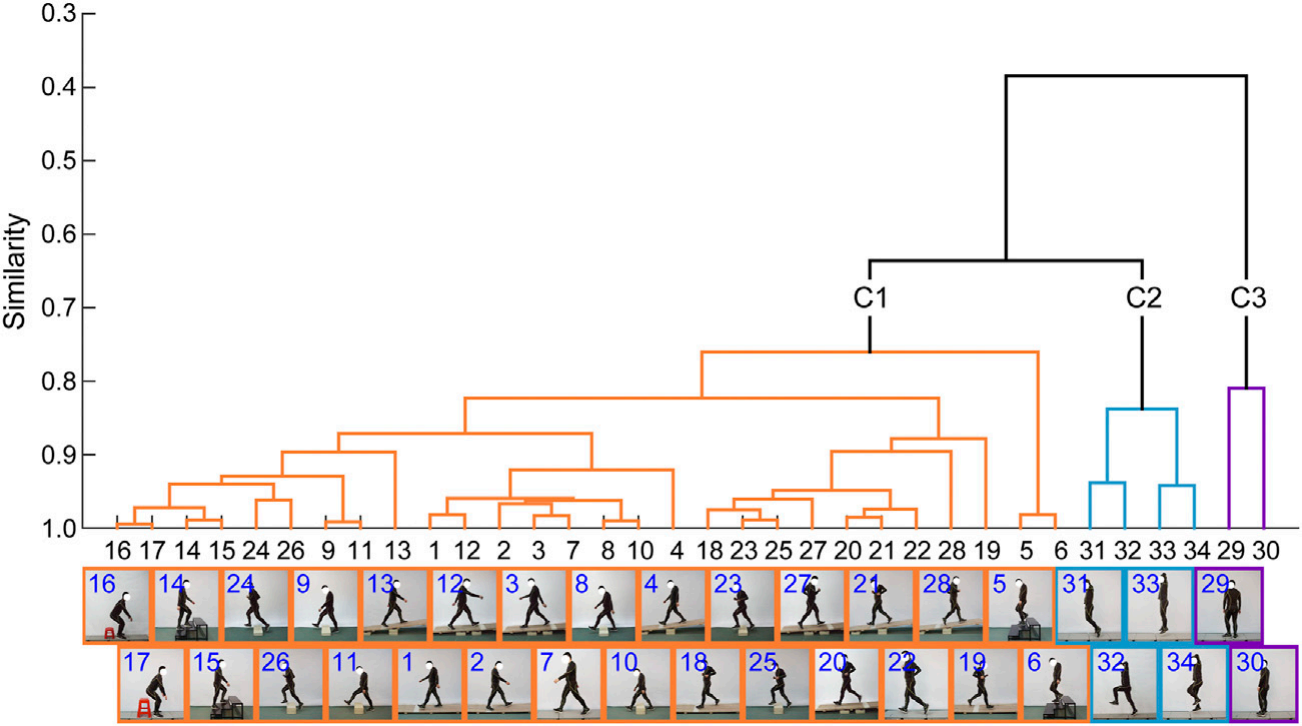
Dendrogram of the lower limb movements. Three clusters (C1–C3) are identified in the various lower limb movements based on the synergistic characteristics of the limb movements.

The similarities among the motor tasks were measured by using the kinematic synergies (Figure 2). Three representative clusters or categories (C1–C3) were identified in the diverse lower limb motor tasks, consistent with the visual inspection of the movement similarities. As shown in Figure 3, the largest cluster (C1), composed of twenty-eight motor tasks, was formed at a similarity level of 0.76, which included walking, running, and sitting-down-standing-up. The other two small clusters (C2 and C3) were also identified. Hopping tasks (involving hopping forward and hopping in place) and turning were the second (C2) and third (C3) clusters, respectively.

### Synergistic Characteristics of the Clusters

**FIGURE 4:**
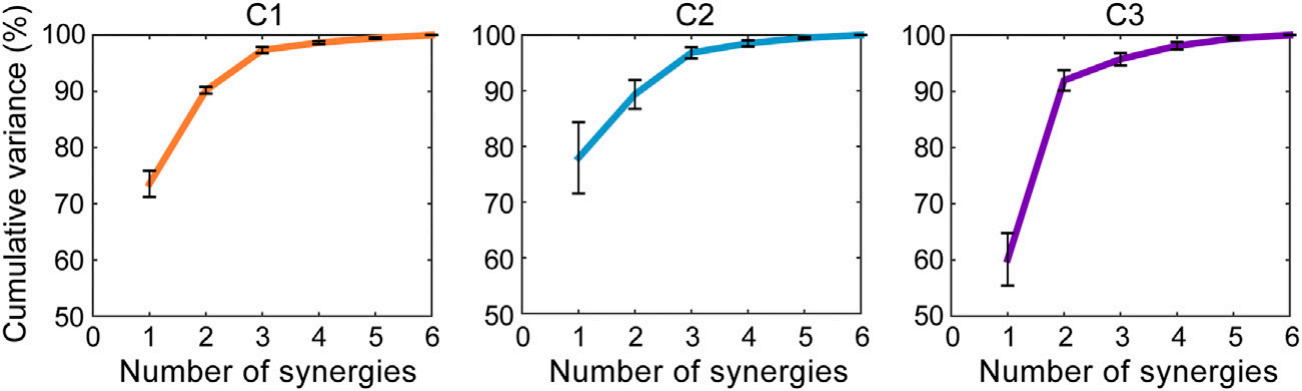
The overall variance of all the tasks in each cluster explained by the core synergies. The core synergies of a cluster represent the common synergistic characteristics of all the motor tasks in the cluster, and are extracted from the data of all the tasks belonging to the cluster. The lines and error bars indicate the means and standard deviations of the cumulative percentage of the overall movement variance explained by the synergies across the subjects (*n* = 9), respectively.

**FIGURE 5:**
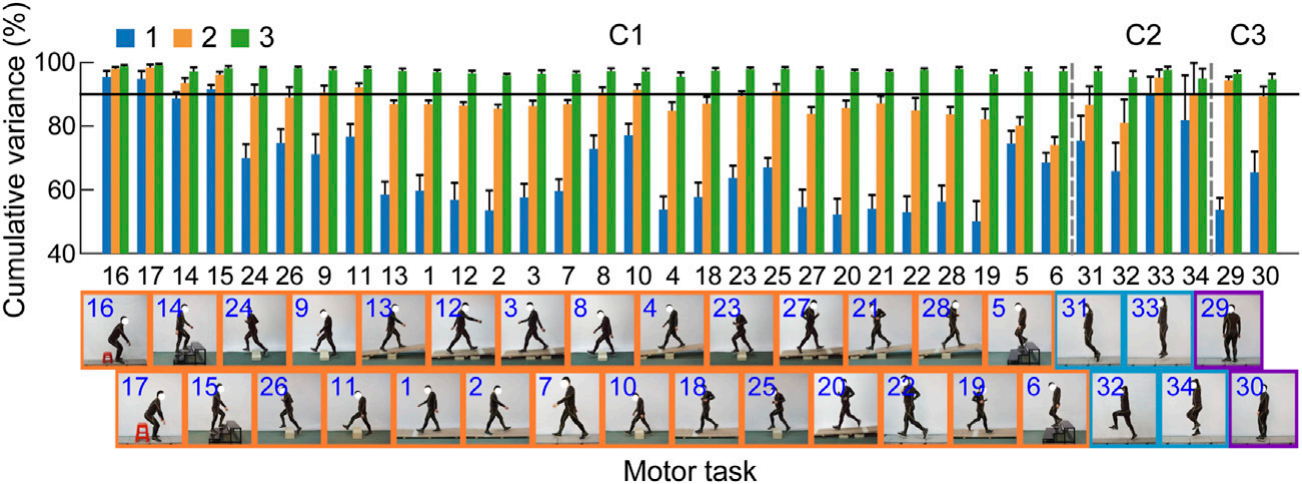
Movement reconstruction of each task by the combination of the core synergies in the clusters (C1–C3). The bar graph depicts the percentage of the total joint motion variance of each task (means and standard deviations across the subjects, *n* = 9) explained by the first (blue), the first two (orange), and the first three (green) core synergies in the three clusters. The reconstruction quality is considered good if the variance explained >90% (black horizontal line).

The core synergies of a cluster representing the common synergistic characteristics of all the motor tasks in the cluster were further extracted. As the results showed, lower limb joint motions could be reconstructed by combining a small number of core synergies in each cluster (Figures 4, 5). The first three synergies accounted for most of the overall movement variance of all the tasks in each cluster (>90%; 97.34 ± 0.53%, 96.83 ± 0.99%, 95.70 ± 1.11% in C1, C2, and C3, respectively; Figure 4). Meanwhile, in each cluster, the limb movement patterns of each task could also be well reconstructed by the first three core synergies (>90%; range: 95.57 ± 1.34% to 99.13 ± 0.51% in C1, 94.93 ± 3.13% to 97.63 ± 1.15% in C2, 94.72 ± 1.69% to 96.41 ± 0.98% in C3; Figure 5).

**FIGURE 6:**
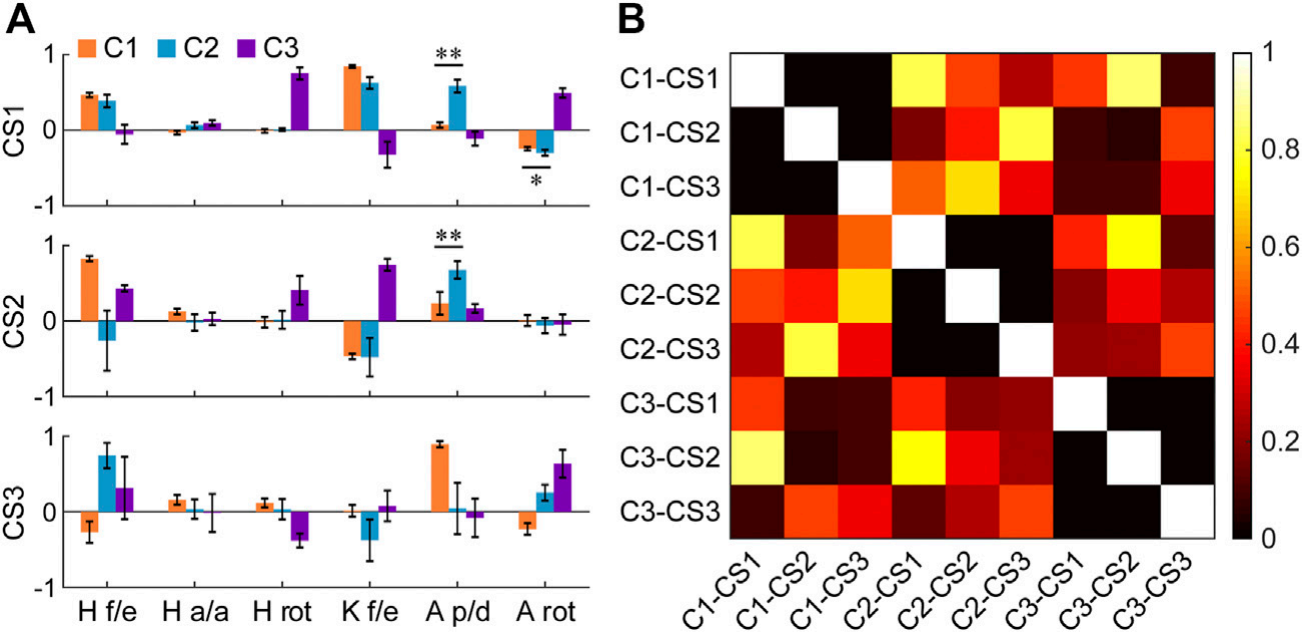
**(A)** Core synergies of the three clusters (C1–C3). The bars and error bars indicate the means and standard deviations across the subjects (*n* = 9), respectively. Before averaging, the direction of the core synergies in a few subjects is adjusted (i.e., reversed) when these synergies are not consistent with corresponding reference synergies in a reference subject (i.e., when the scalar product between the synergy needing to be adjusted and the reference synergy is less than zero). Flexion, adduction, internal rotation, and dorsiflexion are defined as positive values. *p < 0.01, **p < 0.001 (two-tailed paired *t*-test). Abbreviations: CS1: the first core synergy; CS2: the second core synergy; CS3: the third core synergy; H f/e: hip flexion/extension; H a/a: hip adduction/abduction; H rot: hip rotation; K f/e: knee flexion/extension; A p/d: ankle plantarflexion/dorsiflexion; A rot: ankle rotation. **(B)** Similarity matrix of these core synergies (means across the subjects, *n* = 9). The similarity between two synergies is quantified by the absolute value of their scalar product. Two synergies are considered significantly similar if their similarity >0.7. Each of the abbreviations (from C1-CS1 to C3-CS3) represents a core synergy of a cluster. For instance, C1-CS1 represents the first core synergy of the cluster 1.

The primary core synergies of the three clusters showed the movement characteristics in each cluster (Figure 6A). The first core synergies (CS1) of C1 and C2 were similar (>0.7; similarity = 0.82 ± 0.07; Figure 6B) and were characterized by the coordinated movement between hip flexion and knee flexion (or between hip extension and knee extension; average weightings >0.25; Figure 6A). However, in C2, ankle plantarflexion/dorsiflexion and rotation also had noticeable weightings in the CS1 (average weighting = 0.58 and 0.30, respectively), different from the CS1 of C1 (*p* < 0.001 and = 0.004, respectively). In C3, it had a special CS1 compared with the other two clusters, which was characterized by the coordinated movement among hip rotation, knee flexion/extension, and ankle rotation.

For the second and third core synergies (CS2 and CS3), the three clusters further showed respective specific synergistic characteristics. The CS2 of C1 was the coordinated movement between hip flexion and knee extension (or between hip extension and knee flexion), and hip flexion/extension had a larger weighting (*p* < 0.001). The CS2 of C2 was mainly characterized by the coordinated movement among hip flexion, knee flexion, and ankle plantarflexion. Similar to the CS1, ankle plantarflexion/dorsiflexion had a significant weighting (0.68) in the CS2 of C2, different from the CS2 of C1 (*p* < 0.001). In C3, the CS2 was the coordinated movement among hip flexion, hip internal rotation, and knee flexion, similar to the CS1 of C1 and C2 (similarity = 0.85 ± 0.11 and 0.75 ± 0.11, respectively; Figure 6B). Obviously, a primary difference among these similar synergies was the difference in the rankings of their contribution rates in the three clusters (according to the order of the variance explained by the synergies). For the CS3, C1 showed the coordinated movement between hip extension and ankle dorsiflexion, and ankle plantarflexion/dorsiflexion had a larger weighting (*p* < 0.001). The CS3 of C2 was the coordinated movement among hip flexion, knee extension, and ankle internal rotation, which was different from the CS3 of C1 and C3 (similarity = 0.35 ± 0.21 and 0.46 ± 0.26, respectively; Figure 6B), but similar to the CS2 of C1 (similarity = 0.80 ± 0.13). In C3, the CS3 was the coordinated movement among hip flexion, hip external rotation, and ankle internal rotation, which was also different from the other clusters.

## Discussion

In this study, we investigated the quantitative similarity measure and hierarchical categorization of the diverse lower limb movements. Different types of lower limb movements (including cyclical and non-cyclical ones) were well described by a unified movement descriptor, namely, the kinematic synergies, which represent kinematic control strategy in the execution of the limb movements. Based on synergistic characteristics, the similarities among the lower limb movements were quantitatively measured, and three primary homogeneous clusters (C1–C3) were identified within the diverse lower limb movements. The existence of the clusters suggests a numerical categorization model for the human lower limb movements. To our knowledge, this categorization is also the first quantitative categorization for a variety of human lower limb movements to date.

Our categorization establishes a hierarchical structure for the many lower limb movements and divides them into three representative sub-motion categories. By this division, our results provide new insights into the formation mechanisms of the lower limb movements in physiology. The first category (C1), as the largest one, is composed of walking and running under various ground conditions and sitting-down-standing-up. The existence of C1 suggests that similar control strategies are adopted by humans when they perform these walking, running, sitting-standing tasks. This finding supports that humans simplify the generation of a variety of lower limb movements (including diverse gait modes and interaction styles with the environment) by reusing the same basic motor synergies, without the need to develop new synergies *de novo* for each movement. Through this conservation of the kinematic synergies, the seemingly daunting task of achieving the many movements with diverse motor task-related and environmental constraints can be completed in an effective and simple manner. Likewise, the control of motor tasks is also simplified by employing similar synergies in each of the other two categories (C2 and C3). On the other hand, the existence of the three different categories also suggests the flexibility and plasticity of the human motor system. In order to achieve some category-specific behavioral goals or learn several novel skills, humans can also develop new motor synergies. Overall, these findings suggest the coexistence of the conservation and augmentation of the motor synergies underlying the generation of the lower limb movements.

The limb movement patterns of the motor tasks in each category can be effectively generated by the combination of three core synergies. In C1, the coordination of hip and knee flexion/extension plays an important role in the generation of the limb movements, as indicated by the coordinated movements between hip flexion and knee flexion (or between hip extension and knee extension) in its CS1 and between hip flexion and knee extension (or between hip extension and knee flexion) in its CS2. Moreover, the CS3 with the maximum weighting in ankle plantarflexion/dorsiflexion implies that the control of the ankle joint motion is also critical in C1, consistent with the notion that limb endpoint control requires accurate control of the ankle joint motion in human locomotion (Ivanenko et al., 2007). For C2, similar to C1, the coordination of hip and knee flexion/extension also contributes to the formation of the limb movement patterns, as indicated by the synergistic characteristics in the CS1–CS3 of C2. However, different from C1, the main coordination manners adopted by C2 is the coordination of ankle plantarflexion/dorsiflexion with hip and knee flexion/extension. Ankle plantarflexion/dorsiflexion has greater weightings in the CS1 and CS2 of C2 than in the CS1 and CS2 of C1 (Figure 6A). In particular, the coordination among ankle dorsiflexion, hip flexion, and knee flexion (or among ankle plantarflexion, hip extension, and knee extension; the CS1 of C2) is in line with the power transfer mechanism of biarticular muscles among the hip, knee, and ankle joints in hopping (Junius et al., 2017; Schumacher et al., 2020). Through the coordination of the joint motions and the action of biarticular muscles (close to isometric contraction, that is, almost zero contraction velocity), the power can be effectively transported from the hip and knee joints to the ankle joint (or from the ankle joint to the hip and knee joints). In this way, the power demand on the ankle joint that may exceed the capability of ankle joint muscles can be met by the power from the hip and knee joints in hopping. Likewise, this power transfer may also exist between the hip and knee joints *via* their coordination in walking and running tasks (i.e., C1) (Neumann, 2010; Junius et al., 2017). For C3, the existence of the category composed of only turning tasks is consistent with the specific behavioral goal of turning. Different from the other lower limb movements included in C1 and C2 with the aim to push the body forward, upward, or downward, the goal of turning is to rotate the whole body (Ivanenko et al., 2007; Akiyama et al., 2018). This difference is also well characterized by the unique synergistic characteristics of C3 (the coordinated movements between hip and ankle rotations). Taken together, the three categories employ their respective unique synergies.

**FIGURE 7:**
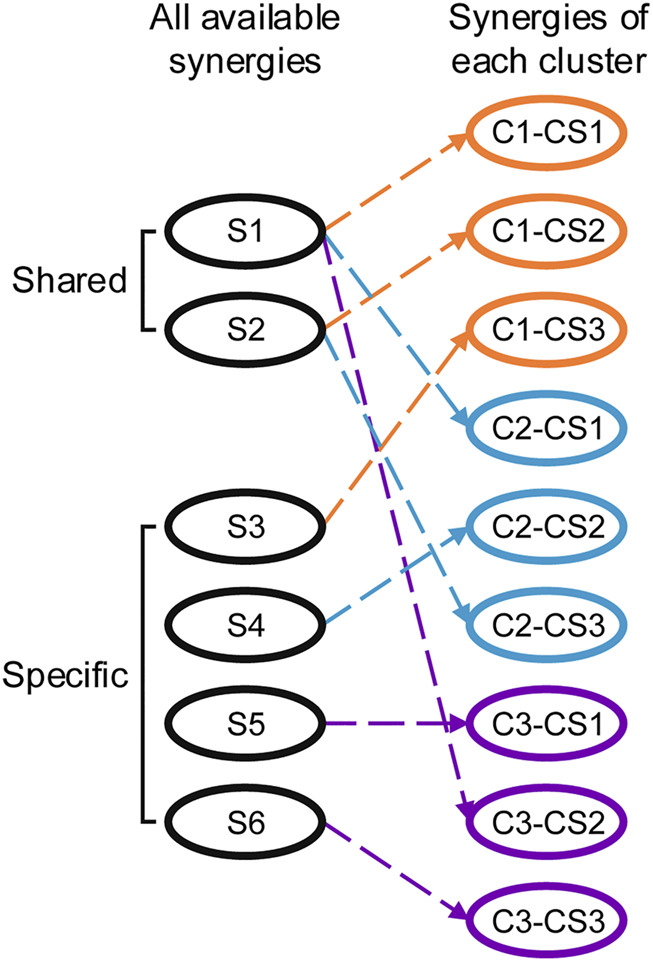
Typical and available synergies employed by humans to achieve the various behavioral goals of the clusters. Shared and specific synergies exist in the three clusters and are used to meet cluster-independent and cluster-dependent behavioral demands, respectively.

Besides the uniqueness of each category, more interestingly, high similarities are also found among some of the synergies of the three categories. In total, there may be a total of six synergies available for the three categories (Figure 7). Two of the six synergies are shared across the categories, and each of the other four synergies is exclusive to a category. Meanwhile, as indicated by our results, the shared synergies also show subtle but significant changes in the weightings of a few joint motions and changes in importance in the three categories. For instance, the second shared synergy (S2) is the second synergy in C1 (C1-CS2), but the third synergy in C2 (C2-CS3). Overall, these findings further suggest that humans can achieve various behavioral goals rapidly and effectively by retaining, fine-tuning, and augmenting the collections of pre-existing motor synergies.

Our categorization also provides inspiration for the studies related to the lower limb movements, such as the motor function assessment and treatment planning in rehabilitation and the development of artificial limbs in robotics (Papageorgiou et al., 2019; Stival et al., 2019). In these studies, the complex question of learning the characteristics of the various lower limb movements can be solved by dividing the many movements into three small, homogeneous, and manageable sub-motion categories. The motor behaviors within one of the three categories which have shared movement strategies can be modeled and analyzed in the same manner. On the basis of the characteristics of each category, customized and standardized rehabilitation programs can be formulated for each of the three categories. The motor function of the lower limb can also be effectively assessed in the process of rehabilitation treatments. Similarly, in robotics, an effective method that can be used to improve the functionality of the artificial limbs reproducing the lower limb movements will be to develop specific mechanical systems for each category or modular/dynamic control systems based on the movement categories. Meanwhile, the core synergies of each category also provide references for the comparison between the human and artificial limbs, which can further accelerate the development of better artificial limbs. In addition, the existence of the three different categories also suggests the strategy of prioritizing part of the categories according to practical demands in rehabilitation and robotics (e.g., feasibility of motor recovery or functional requirements of the artificial limbs).

As a descriptor to characterize the lower limb movements, the kinematic synergies represent the coordination strategy in the movement process and provide a basis for measuring the similarities among different types of lower limb movements in this study. Based on this, a categorization composed of three distinct movement categories is successfully built, and task-independent and task-dependent synergies are also revealed in human locomotion. In fact, in addition to the lower limb movements, kinematic coordination has also been found in the other limb movements, such as hand grasps (Xiong et al., 2016; Jarque-Bou et al., 2019) and upper limb movements (Schuetz and Schack, 2013; Liu et al., 2018). Consequently, this synergy-based movement representation has the potential to be used to quantify the similarity of the other limb movements. Moreover, synergies have also been observed at kinetic or muscular levels (Giszter, 2015; Scano et al., 2017). This means that our measure method of movement similarity can also be extended by taking into account the kinetic and muscle synergies in the future, which will provide more detailed and complementary information regarding the generation of the limb movements.

There are some limitations to the study. First, to identify the sub-motion categories underlying the diverse lower limb movements, here we selected thirty-four typical motor tasks, which to some extent represent the versatile motor ability of the lower limb (Kuehne et al., 2011; Mandery et al., 2016). However, it is well known that humans can move in an infinite number of ways in daily living. In this case, while it can be expected that our methodology will also be able to provide beneficial guidance for the characterization of the motor tasks that are not explored in this study, further analyses need to be performed in the future. Likewise, in practical applications, researchers may also pay attention to only part of the motor tasks we have studied. For these tasks (e.g., only walking and running), our methodology must be adapted in order to obtain a partial categorization, and subtler movement characteristics may be further uncovered. For example, the dendrogram in Figure 3 shows that walking downstairs may require somewhat different kinematic synergies from the other walking tasks and trends to form a single category. Second, the number of participants included in this study was small (*n* = 9). In the future, the sample size should be enlarged for further verification of our methodology. Third, only healthy adults were considered in this study. Future work is necessary to evaluate the efficacy and versatility of our methodology in characterizing the other human gaits (including gaits of children or older adults or diverse pathological gaits). Fourth, here we only analyzed the joint kinematics without considering the joint kinetics or muscle activities. As mentioned above, further studies extending our methodology by considering the kinetic and muscle synergies are necessary, which can provide new insights into the formation mechanisms underlying the lower limb movements.

## Conclusion

This study proposes a general framework for measuring the similarities among the limb movements based on the kinematic synergies and establishes a quantitative and hierarchical categorization for the lower limb movements. Three main categories are identified. In each category, the motor tasks can be well reconstructed by combining three core synergies, and shared synergies are also found across the three different categories. The coexistence of synergies shared across the movements and categories and synergies for specific categories thus suggests that there exists an effective strategy for humans to simplify the formation of the various lower limb movements by retaining, fine-tuning, and augmenting initial collections of the kinematic synergies. Besides providing inspiration for understanding human movements, the categorization consisting of manageable and homogeneous categories can also be expected to facilitate the motor function assessment and treatment planning in rehabilitation and the development of better artificial limbs in robotics, which deserves to be investigated in the future. Moreover, our proposed approach also provides a means to quantify the degree of similarity and build a hierarchical description for the other human limb movements, even the movements of other animals.

## Data Availability

A publicly available dataset was analyzed in this study. This data can be found here: Dryad Digital Repository, https://doi.org/10.5061/dryad.wdbrv15n9.
